# Genotypes, mutations, and viral load of hepatitis B virus and the risk of hepatocellular carcinoma: HBV properties and hepatocarcinogenesis

**Published:** 2011-02-01

**Authors:** Qi Zhang, Guangwen Cao

**Affiliations:** 1 Department of Epidemiology, Second Military Medical University, Shanghai, China

**Keywords:** Hepatocellular carcinoma, Hepatitis B virus, Genotype, Mutation

## Abstract

Chronic infection with hepatitis B virus (HBV) is the major risk factor for hepatocellular carcinoma (HCC) worldwide. Ten HBV genotypes (A-J) have been discovered so far. Genotypes B and C are endemic in East and Southeast Asia. Genotype C HBV is associated with increased risks of cirrhosis and HCC. Genotype B (B2) is associated with the development of HCC in non-cirrhotic patients younger than 50 years and with relapse of HCC after surgical treatment. It is also associated with earlier hepatitis B e antigen seroconversion than genotype C. High HBV load is independently associated with the occurrence and post-treatment recurrence of HCC. Different genotypes have distinct patterns of mutations. Viral mutations in the core promoter region and in the preS region are frequently found to be significantly associated with an increased risk of HCC. These mutations often occur before the onset of HCC and accumulate during the progression of chronic HBV infection. Multiple such mutations are more frequent in patients with HCC and are specific for HCC. HBV subgenotypes, viral mutations, and viral load can be used for the prediction of HCC. Early identification of HBV-infected individuals who will eventually develop HCC will help to develop active prophylactic protocols to reduce or delay the occurrence of HCC.

## Epidemiology of hepatocellular carcinoma

Primary liver cancer is the third-most-common cause of cancer death worldwide [1], and hepatocellular carcinoma (HCC) accounts for 85-90% of these cancers. Worldwide, between 500,000 and one million new HCC cases are diagnosed each year, with an age-adjusted annual incidence of 14.9 per 100,000 in men and 5.5 per 100,000 in women [2][3]. More than 80% of the cases occur in East Asia (age-adjusted annual incidence 35.2-48.8 per 100,000 in men and 11.6-13.3 per 100,000 in women) and Sub-Saharan Africa (age-adjusted annual incidence 28.5-39.7 per 100,000 in men and 12.2-14.6 per 100,000 in women), where hepatitis B virus (HBV) infection is endemic. China alone accounts for 55% of all HCC patients [1]. In contrast, the incidence of HCC is low in North and South America, Western and Northern Europe, and Oceania, whereas the median incidence rate of HCC is reported in Southern Europe [4]. In Iran, the annual incidence of HCC is low (0.2 per 100,000, sex- and age-adjusted), possibly because of the low prevalence of chronic HBV infection (CHB) [5][6].Established risk factors for HCC include chronic infection with HBV and/or hepatitis C virus (HCV), old age, male sex, aflatoxin exposure, alcoholic abuse, diabetes, nonalcoholic fatty liver disease (NAFLD), hemochromatosis, and various host genetic factors. The pattern of major risk factors for HCC varies from country to country. In developing countries, CHB with or without aflatoxin exposure is responsible for the most cases. In developed countries, however, over 90% of HCC cases occur in the background of cirrhosis caused by chronic HCV infection and alcohol abuse [7][8]. In developed countries, people get infected by HBV mainly through sexual and other horizontal transmission in adulthood, with more than 90% of HBV cleared spontaneously. In developing countries, in contrast, HBV is usually transmitted from mother to child in the perinatal period, and around 90% of infected newborns develop CHB [4]. CHB is the most important risk factor for HCC in the world. It contributes to more than 50% of HCC cases worldwide and 70-80% of HCC cases in the high-endemic regions of HBV. The annual incidence of HCC among people with CHB ranges from 400-800 per 100,000 in men and from 120-180 per 100,000 in women. The relative risk of HCC among people infected with HBV ranges from 5-49 in case-control studies and from 7-98 in cohort studies [9]. Standard HBV vaccination programs decrease the prevalence of HCC among the vaccines [10]. However, the influence of such programs on the incidence rate may not be obvious for at least two decades due to the high prevalence of CHB and prolonged latency to HCC development [9]. Recently, a decreasing trend in the incidence of HCC in high-risk areas has been reported. The vaccination of all newborns against HBV and a shift in diet from corn to rice in some parts of China, leading to decreased exposure to aflatoxin, may be the major reasons for this decline in incidence [4]. In contrast to the decline in developing countries, the incidence of HCC has been rising in some developed countries, including the United States, Italy, the United Kingdom, Canada, and Australia [2]. This increase is mainly attributed to a greater prevalence of HCV infection in these areas and the rising rates of obesity and NAFLD [10]. Immigration from HBV-endemic countries may also contribute to the increasing incidence of HCC. The incidence of HCC increases with age, reaching a peak among those aged 50-65 in HBV-endemic areas and beyond age 65 in non-endemic areas. It was rare for people in western countries to develop HCC before 50 years old. But in the last two decades, there has been a shift of incidence to patients age 40-60 [11], possibly because of an increase in HBV-infected individuals in these countries. With the exception of study populations with extremely low prevalence of HCC, men are 2-4 times more often affected by HCC than women, which may be caused by androgenic hormones [12]. Host genetic predispositions, such as single nucleotide polymorphisms of specific inflammatory factors, have recently been reported to be risk factors for HCC [13][14][15]. This may help explain why China accounts for only 1/4-1/3 of all HBV infections, but more than 50% of all HCC cases. Despite major efforts to improve diagnosis and treatment, HCC still carries an extremely poor prognosis, leading to 600,000 deaths globally each year [2]. The median survival for HBV-related HCC is less than 16 months, and the five-year survival rate ranges from 15-26% [9]. The most effective treatments for HCC are surgical resection, liver transplantation, and radiofrequency ablation [16]. However, these treatments are available to only a low percentage of patients, recurrence rates are still high. Thus, it is important to understand the mechanisms of HBV-induced hepatocarcinogenesis and identify the predictors for HCC. Early identification of HBV-infected individuals who will eventually develop HCC is important for developing effective prevention methods, active surveillance, and novel treatment options. In this review, we mainly focus on the HBV properties and their associations with the risk of HCC.

## Viral replication and the association with HCC

**Figure 1:**
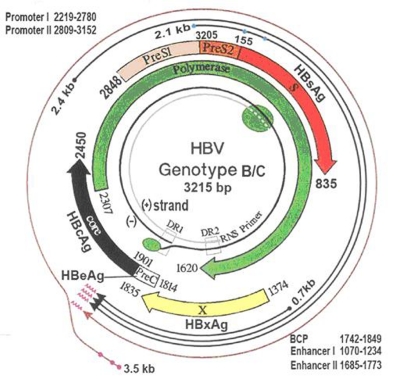
Diagram for HBV genomic structure

HBV belongs to hepadnaviridae, a family of enveloped viruses with an incomplete double-stranded DNA genome of 3.2 kb (Figure 1). The HBV genome contains four overlapping open reading frames, which encode the three hepatitis B surface antigens (HBsAg) (encoded by the S gene, preS2/S genes and preS1/preS2/S genes), hepatitis B e antigen (HBeAg) and hepatitis B core antigen (HBcAg) (encoded by preC/C genes), viral polymerase (encoded by P genes), and a multifunctional nonstructural protein called X (encoded by the X gene). The preS region (nucleotides [nt] 2854-155), which consists of preS1 and preS2, overlaps a region encoding the polymerase gene. The enhance II (Enh II, nt.1636-nt.1744) and basal core promoter (BCP, nt.1751-nt.1769) regions overlap with the X gene (nt.1374-nt.1835). The overlapping structure of the coding regions makes the utility of the HBV genome more than 150%. The BCP, which is regulated by Enh II and to some extent by Enh I, controls the transcription of precore mRNA and core mRNA and viral generation [17]. The precore region encodes the precore protein, which is processed in the endoplasmic reticulum to produce secreted HBeAg. HBeAg is clinically used as an indicator of active viral replication and has, along with viral load, been associated with an increased risk of HCC in a prospective study [18]. A HBV DNA load higher than 1×104 copies/mL is an important predictor of the development of HCC in asymptomatic HBsAg carriers (ASCs), independent of HBeAg, alanine aminotransferase (ALT) level, or cirrhosis [19]. Viral load is associated with an increased risk of cirrhosis in HBeAg-negative HBV-infected individuals [20]. The interval from diagnosis of cirrhosis to development of HCC among high-HBV DNA individuals is much shorter than that among persistently low-HBV DNA individuals [21]. This evidence indicates that high viral load is a reliable marker of high risk of HCC in HBV-infected individuals. High viral load and active hepatitis can also be found in some HBV carriers who have already developed anti-HBe antibody. There are two well-defined mutations reported to be associated with HBeAg expression. One is a G-to-A mutation at nt.1896 (G1896A) in the precore region, which induces a stop codon and cessation of HBeAg expression. Another is the double mutation A1762T/G1764A in the BCP region, which decreases HBeAg production by 70% through suppressing the transcription of precore mRNA [22][23]. Thus, HBeAg status and anti-HBe status are less reliable markers than HBV DNA level for viral replication or the risk of HCC. High viral load is also an important risk factor for post-treatment recurrence of HCC, irrespective of the pattern of recurrence [24]. Large prospective studies are needed to see if antiviral therapy can reduce or postpone the occurrence of HCC or improve the tumor-free survival rate after curative resection in HBV-related HCC patients with high viral load.

## HBV genotypes and their associations with HCC

**Figure 2:**
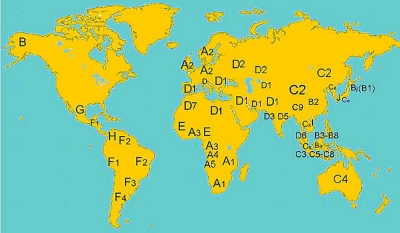
Global distribution of HBV genotypes and subgenotypes. A-J, HBV genotypes A-J; Ba=B2; Bj=B1; Ce=C2; Cs=C1.

According to a sequence divergence greater than 8% in the entire HBV genome, 8 genotypes (A-H) have been identified [25], and two additional genotypes, provisionally designated as genotype I and genotype J, have been isolated in Vietnam and Japan, respectively [26][27]. HBV genotypes can be further divided into subgenotypes if the divergence in the whole genome is between 4-8%. HBV genotypes and subgenotypes have distinct geographical distributions (Figure 2) [25][28][29][30]. Genotype A is endemic in southern and western parts of Africa (subgenotypes A1, A3-A5) and western and northern parts of Europe (A2). Genotypes B and C are endemic in eastern and southeastern Asia. Genotype D is endemic in the Mediterranean area, the Middle East, and other parts of western Asia. Genotype E is restricted to West Africa and genotype F is frequent in Central America. Genotypes G and H have been found in South America. Our recent community-based study found that in Mainland China subgenotypes B2 (27.3%), C1 (10.7%), and C2 (58.0%) are predominant [31]. We found that the compositions of subgenotypes B2, C1, and infection with multiple HBV genotypes (genotype mixture) increase from the north to central south of China, and is associated with an increasing prevalence of HBsAg. The distribution of HBV genotypes and subgenotypes may help in determining disease burden. HBV genotypes and subgenotypes have been shown to differ with regard to HBeAg seroconversion, clinical outcomes, prognosis, and antiviral responses. However, the role of HBV genotypes in HCC risk is still controversial [32]. Sample size, predominance of one genotype in one population, study design and other confounding factors are issues for both positive and negative findings. In Mainland China, seropositive HBeAg is more common in subgenotype B2-infected individuals younger than 35 years, rather than in those older than 35 years, as compared with individuals infected with subgenotype C2. The same is true for HBV DNA level between individuals infected with subgenotype B2 and subgenotypes C1/C2 [31]. Genotypes A, B, D, and F are associated with earlier spontaneous HBeAg seroconversion than genotype C, and HBeAg seroconversion confers favorable long-term outcomes [31][33][34][35]. We have recently demonstrated that subgenotype B2 is more likely to be associated with acute HBV, while genotype C (subgenotype C2) is more likely to cause CHB than subgenotype B2 following an acute infection course [36]. Studies in East Asia have demonstrated that genotype C CHB is more likely than genotype B to cause cirrhosis and HCC [37][38][39][40]. Genotype C CHB is associated with an increased risk of HCC in cirrhotic patients over 50 years, whereas infection with genotype B (B2) has been found to be associated with the development of HCC in non-cirrhotic young patients and relapse of HCC after surgical treatment [40][41][42]. High viral load and genotype C have an additive role in increasing the risk of HCC [40]. Although subgenotypes C1 and C2 are associated with an increased risk of HCC, only subgenotype C2 is independently associated with HCC [43]. Genotype mixture, which is frequently found using multiplex PCR method [44], is often associated with higher viral load and a more severe course of liver disease than genotype C alone [41]. Subgenotype B1 (Bj) is restricted to Japan, while subgenotype B2 is endemic in most Asian areas. In Japan, the average age of HCC patients with subgenotype B1 is older than those with genotype C. Individuals with subgenotype B1 are frequently negative for HBeAg, have lower ALT levels, lower HCC occurrence, and a better prognosis [45]. Genotype D has been associated with more severe liver disease than genotype A and may predict the occurrence of HCC in young patients [46]. Different genotypes have shown different responses to interferon-alpha (IFN-α) treatment. Compared to genotype C, genotype B has a higher rate of response to IFN-α treatment in HBeAg (+) CHB, and genotypes E, F, and H appear to be more sensitive to IFN-α than genotype G [47][48]. Furthermore, genotype A is more sensitive to interferon than genotype D [49]. Taken together, subgenotype B2 is associated with high viral load and HCC in young patients, with less frequency of cirrhosis, and is also associated with recurrence of HCC after surgery, possibly because of high viral load. Subgenotype C2 is more likely to be associated with persistent HBV infection following an acute course, and is associated with a high percentage of HCC in cirrhotic patients. IFN-α treatment is effective for patients with genotype A or B. There is rare evidence to show the association of other genotypes with the risk of HCC and response to treatment because a unique HBV genotype prevails in many areas. International collaboration will help in elucidating the association of all HBV genotypes with the risk of HCC.

## HBV mutations and their associations with HCC

Because the reverse transcriptase lacks a proofreading function, HBV exhibits a higher mutation rate than other DNA viruses. HBV mutants can show enhanced virulence, resistance to antiviral treatment, facilitated cell attachment or alteration of epitopes important in the host immune response [50]. Mutations might be generated during HBV-induced pathogenesis or antiviral treatment. Random viral mutation and directional selection by host immunity confer a distinctive mutation pattern of HBV in each liver disease. The HCC-associated mutations often occur in the Enh II/BCP/precore and preS regions. Takahashi et al. [51] first identified HBV mutations through the comparative analysis of full-length HBV isolates (95% genotype C) from 40 HCC patients, and found that mutations in the preS2 region, A1762T, G1764A, and T1753C/A mutations in the BCP region, and G1613A and C1653T mutations in the Enh II region were more frequent in HCC patients. Numerous associations between HBV mutations and risk of HCC have since been reported [52]. The different HBV genotypes display distinct mutation patterns in the preS and Enh II/BCP/precore regions [31]. To elucidate the association of HBV mutations with liver diseases, it is essential to clarify wild-type patterns in individuals without clinical liver diseases. However, previous studies have not shown the wild-type pattern of a given HBV genotype and the genotype-specific pattern of mutation as well. At the early stage of HBV infection, the wild-type form facilitates transmission from mother to child. Children whose mothers carry mutants were mostly found to be infected with just the wild-type form of the same viruses, suggesting that the HCC-associated mutants (e.g., preS deletion mutants and A1762T/G1764A mutants) may not transmit from mother to child [53][54]. In the HBeAg-positive period, viral load is high and immune selection is week. Immune selection may increase during HBeAg seroconversion. HBeAg negativity has been associated with the presence of mutations in the BCP/precore regions. The HBeAg-negative variant has a selective advantage over wild-type HBV within the livers of patients with CHB during an immune response [55]. We have recently defined wild-type nucleotides at the hotspots (=10% mutation rate in the entire subjects) in the Enh II/BCP/precore or the preS region of genotypes B and C from HBeAg-positive "quasi-immunotolerant" ASC, and then evaluated the associations of these mutations with HCC [56][57][58][59]. This strategy has revealed a group of important mutations that are associated with the risk of HCC:

### 1) Mutations in the Enh II/BCP/precore region

We and others have found that C1653T, T1753V, A1762T/G1764A, T1674C/G, and T1766/A1768 are associated with an increased risk of HCC [52][56][60]. Frequencies of T1674C/G, C1653T, T1753V, and A1762T/ G1764A were more than 30% in patients with HCC, which make clinical examination feasible and clearly discriminate HBV-infected patients with and without HCC [56]. A recent study showed that G1899A, C2002T, A2159G, A2189C, and G2203A/T in the precore/core gene are closely related to HCC [61]. We have demonstrated that mutations at nt.1653, nt.1674, nt.1719, nt.1753, nt.1762, and nt.1846 in genotype C are associated with an increased risk of HCC compared with ASCs and patients with CHB. These associations are only found in HBeAg-negative individuals. Interestingly, C1673T, A1726C, A1727T, C1730G, T1768A, C1773T, and C1799G are associated with an increased risk of cirrhosis compared with patients with CHB, whereas these mutations are inversely associated with HCC compared with patients with cirrhosis [56]. Recent prospective studies have demonstrated that A1762T/G1764A occurs up to 10 years before the onset of HCC and is a useful predictor of HCC [62][63][64]. Importantly, of the HBV mutations independently associated with an increased risk of HCC, haplotypic carriages with two or more mutations, rather than a single one, are associated with an increased risk of HCC [56]. C1653T, T1674G/C, T1753V, A1762T, and G1764A mutations lead to amino acid (aa) transitions at aa94 (H-to-T), aa101 (S-to-A/P), aa127 (I-to-T/N/S), aa130 (K-to-M), and aa131 (V-to-I), respectively. In addition, A1762T introduces a translation initiation site ATG at the C-terminal of X protein. HBV X mutations, especially the C-terminal-deleted X protein, have been frequently found in HCC specimens [64][65][66]. Further, the Enh II/BCP/precore is the regulatory region of HBV. The HCC-associated mutations lead to generation of HFH-2-, HNF-3b-, Skn-1-, Cdx-1-, cap-, and Oct-1-binding sites, which might represent novel virus-host interactions related to development, viral replication, proto-oncogene activation, and carcinogenesis [57]. Thus, HBV genotype-associated mutations in the Enh II/BCP/precore region might be selected by HBV-host interaction during CHB and, in turn, promote hepatocarcinogenesis. It is also possible that the mutated X protein trans-activates host oncogenes responsible for the development of HCC or that trans-activators encoded by some oncogenes select the specific HBV mutations during HBV-induced hepatocarcinogenesis.

### 2) Mutation in the preS region

The preS1 and preS2 regions contain several epitopes for T and B cells, thus playing an essential role in the interaction with host immune responses [17]. The most common mutation in the preS is preS deletion [52], leading to an inefficient immune response. The preS deletion also decreases the expression of surface proteins, resulting in intracellular accumulation of HBV envelope proteins and viral particles, formation of ground-glass hepatocytes, endoplasmic reticulum stress, and oxidative DNA damage [17]. These changes eventually lead to hepatocarcinogenesis. There is no evidence that the preS mutation can transmit from mother to child, so preS mutations are generated during the progression of CHB, especially in patients treated with IFN [30][53]. The preS deletion has a higher prevalence in genotype C than genotype B, and is reported to be closely associated with occult HBV infection and HCC development [67][68][69]. Our recent studies have demonstrated that T53C, preS2 start codon mutation, preS1 deletion, and novel mutations including C2964A, A2962G, C3116T, and C7A are associated with an increased risk of HCC, while a novel mutation C105T in preS2 region is inversely associated with the risk of HCC [57][58][59]. The frequencies of combined preS mutations such as 2964C-3116T-preS2 start codon wild-type-7A, 2964C-3116T-7A-76C, and 2964A-3116T-7C-76A/T are higher in patients with HCC, whereas the haplotypic carriages with a single mutation and other three wildtypes are inversely associated with HCC [58]. C2875A, G2946C, G2950A, G2951A, A2962G, C2964A, T3066A, T3069G, C3116T, A3120T, C7A, and T53C are inversely associated with the risk of cirrhosis, whereas these mutations are associated with an increased risk of HCC [56], indicating HCC and cirrhosis might have distinctive processes of immune selection for mutations.

## HBV properties for the prediction of HCC

As mentioned above, evidence from case-control studies and cohort studies have demonstrated that high viral load (>104 copies/mL), genotype C (especially subgenotype C2), and mutations (especially in the Enh II/BCP/precore and the preS regions) are independently associated with increased risks of HCC, indicating that these viral properties can be used for the prediction of HCC in HBV-infected individuals. A1762T/G1764A can be detected up to 10 years before the onset of HCC, and mutations such as the preS deletion and the complex mutations in the BCP regions frequently occur from months to years after the detection of A1762T/G1764A [60]. The frequencies of the preS deletion, C1653T, T1753V, and A1762T/G1764A mutations consecutively increased during the progression from ASCs to HCC, indicating that these mutations accumulate before the diagnosis of HCC [52]. As different mutations have distinctive power to indicate the risk of HCC, only combined mutations rather than a single one in HBV genome can have a predictive value for the development of HCC. These associations are mostly obtained from cross-sectional case-control studies, a study design that is only able to show a statistical relationship. Prospective studies with comparison group(s) are better for demonstrating a causal relationship. In the reported cohort studies, however, HBV properties are mostly documented based on only one time-point. This kind of study design can demonstrate an association between early HBV properties, such as A1762T/G1764A, and the development of HCC. Sequential HBV mutations prior to HCC have been documented in only a very limited number of individuals [60][70]. Sequential examination of mutations in the Enh II/BCP/precore and/or the preS regions might provide more accurate information than cross-sectional data for the prediction of HCC [71]. Risk factors that have been shown to independently predict the development of HCC in HBV-infected individuals include male gender (relative risk [RR] 2.98), increasing age (RR 1.07), high HBV DNA levels (RR 1.28), core promoter mutations (RR 3.66), and cirrhosis (RR 7.31) [72]. Combining these risk factors with clinical parameters such as albumin and bilirubin, score systems have been created to predict the occurrence of HCC with high accuracy [72][73] Thanks to these prediction systems, HBV-infected individuals who will eventually develop HCC might be early identified. Antiviral treatments for these high-risk individuals might reduce or postpone the occurrence of HCC, while frequent examinations may increase early diagnosis and early treatment options. However, there are insufficient data on viral properties and HCC from the areas where genotypes A, D, E, F, and G are endemic. Such data are necessary to expand the applicability of HCC risk analyses.

## Conclusions

HBV genotype/subgenotype, viral load, and viral mutations in the Enh II/BCP/precore and preS regions are closely associated with hepatocarcinogenesis. These viral properties can be used for the prediction of HCC in individuals with CHB. Understanding which HBV-infected individuals are more likely to develop HCC will greatly aid in the prevention and control of this malignancy in a cost-effective manner.
